# The relationship between stigmatisation and quality of life in Ghanaian women and men with fertility problems: mediating role of coping strategies

**Published:** 2021-01-08

**Authors:** FB Van Rooij, HMW Bos, T Gerrits, RA Hiadzi, ES Donkor

**Affiliations:** Research Institute of Child Development and Education, University of Amsterdam, PO Box 15780, 1001 NG, Amsterdam, The Netherlands; Faculty of Social and Behavioural Sciences, University of Amsterdam, PO Box 15509, 1001 NA Amsterdam, The Netherlands; Department of Sociology, University of Ghana, PO.Box LG 65,Legon, Ghana; School of Nursing and Midwifery, University of Health and Allied Sciences, PMB 31 Ho, Volta Region, Ghana.

**Keywords:** coping, infertility, fertility, Ghana, stigma, quality of life

## Abstract

**Introduction:**

Fertility problems may have a devastating impact on the people involved. Specifically, in highly pronatalist settings like Ghana, the personal and social consequences are high. This study focused on the relationship between stigmatisation because of fertility problems and quality of life among Ghanaian women and men, and the possible mediating role of coping strategies.

**Methods:**

Participants (38 women, 11 men) were recruited with the help of a patient organisation and a hospital in Accra. Standardised instruments were used to measure the stigmatisation of having fertility problems, fertility quality of life and coping with fertility problems. Partial Pearson r correlations were conducted, followed by bootstrapped mediation analyses (PROCESS macro).

**Results:**

Stigmatisation was negatively correlated with fertility quality of life, and fertility quality of life was negatively correlated with active-avoidance coping. Active avoidance coping partially mediated the relationship between being stigmatised because of fertility problems and fertility quality of life.

**Conclusions:**

Professionals working with people with fertility problems should pay more attention to how people are coping with experiences of stigmatisation.

## Introduction

Prevalence rates estimate that 15% of Ghanaian women of childbearing age experience fertility problems in that they are not able to conceive a child after one year or more of regular unprotected sexual intercourse with their partner (Donkor and Sandall, 2009). What does it mean for such people in Ghana when they have to deal with fertility problems? To which extent do they feel or perceive to be negatively labelled by the society in terms of status loss, i.e., stigmatisation? (Link and Phelan, 2001; Phelan et al., 2008) Does this influence the way they are coping with their situation of not being able to become pregnant, and in turn what does this mean for their well-being in terms of their quality of life? There is a lack of knowledge about these mechanisms for people who are confronted with fertility problems in a society like Ghana, in which having children is valued as very important (Tabong and Adongo, 2013), despite the government’s longstanding policy to reduce the country’s population and number of children per woman (Kwnakye and Cofie, 2015).

The values Ghanaians attach to having children are multifaceted (Tabong and Adongo, 2013). Ghanaians see their offspring, for example, as a source of joy, and children also play an important role in assisting at home adding to the family income, or to provide assistance and security in old age (Nauck and Klaus, 2007; Tabong and Adongo, 2013). Having children also strongly adds to the status (i.e., social self-esteem) Ghanaian people receive within their families, family-in-law and communities. It is therefore important for them to desire a child. This sentiment is even stronger for women, where birth-giving and becoming a mother is seen as a rite-of-passage into adulthood (Alhassan et al., 2014; Tabong and Adongo, 2013). Continuing the family-lineage is also valued very highly, and people specifically may desire male children (Tabong and Adongo, 2013). Having children also influences the way you are buried and provides membership in the ancestral world after the person’s death (Tabong and Adongo, 2013). Furthermore, people in Ghana stress the importance of having children in order to obey God’s words to procreate (both within Christianity and Islam) (Donkor and Sandall, 2007; Tabong and Adongo, 2013).

Due to the various roles children have and the high value placed upon having children and parenthood in Ghana, the consequences of having problems in becoming pregnant are very high (Alhassan et al., 2014). Qualitative and quantitative studies show that a substantial proportion of the Ghanaian women in these studies who had to deal with fertility problems experience stigmatisation and are labelled as abnormal or incomplete in their daily life (Donkor and Sandall, 2007; Fledderjohann, 2012; Yebei, 2000; Hiadzi and Woodward, 2019). Men are stigmatised as well (Tabong and Adongo, 2013), although to a lesser degree (Fledderjohann, 2012; Tabong and Adongo, 2013).

The experiences of being stigmatised because of having fertility problems also influences the well- being of Ghanaian women with fertility problems. They report high levels of infertility related stress, and a high prevalence of depression (Alhassan et al., 2014; Donkor and Sandall, 2007; Naab et al., 2013). Less is known, however, about the processes modifying the association between stigmatisation and well-being amongst Ghanaian people with fertility problems.

According to the psychological mediation framework (Hatzenbuehler et al., 2013), coping styles (i.e., strategies, thoughts and actions people use to manage a certain stressful situation) (Lazarus, 1993; Lazarus,1998; Lazarus and Folkman, 1984) might play an important role in the association between stigmatisation and well-being. This framework assumes that 1) people experience increased stress from being stigmatised, 2) this increased stress generates dysregulation of inter- and intrapersonal psychological processes, and 3) these processes in turn mediate the relationship between stigma related stress and psychological problems. In line with this psychological mediation framework, studies have shown that coping strategies are such intra-psychological processes that mediate the association between stigmatisation and well-being (Koball and Carels, 2013; Sanjuán et al., 2013). Coping reflects upon how people deal with stressful and difficult situations and includes cognitive, behavioral, and emotional attempts to manage the demands imposed by a stressor (Compas et al., 2001; Lazarus, 1993; Lazarus, 1998; Lazarus and Folkman, 1984).

Studies among Ghanaian women who have had to deal with fertility problem showed that these women used several coping strategies such as drawing on their religion, avoiding certain situations, getting the support of their partner, or becoming economically independent (Donkor and Sandall, 2009). However, whether the coping styles that Ghanaians who are confronted with fertility problems are using mediate the association between being stigmatised and well-being in terms of quality of life, has not previously been studied. We hypothesised that the stigmatisation experienced has an indirect negative effect on quality of life through the coping styles that people are using in the way they are dealing with the fertility problems.

## Methods

### Participants

In total 49 Ghanaian men and women who were experiencing fertility problems in conceiving a child and were visiting a clinic participated in the study by filling in a questionnaire. Demographic characteristics and the fertility status and history of the participants are presented in Table I. The sample consisted of 38 women and 11 men, and all of them were involved in a partner relationship with someone of the other sex. On average the participants were 39.16 years old (SD = 6.37). Their partners were on average 40.51 years old (SD = 5.84). Almost all participants lived in a (large) city (95.9%), and all of them answered to being religious. About two-thirds of the sample reported that they had never been pregnant in their current relationship (or for males that their current female partner had never been pregnant). They had been trying to conceive a child on average for more than 7 years (M = 7.61, SD = 4.70) at the time that the study took place. Of the 18 participants who had experienced a pregnancy in their current relationship, 33.3% reported they had one or more living child(ren). About one third of the sample did not know the reason(s) for their current fertility problems (Table I).

**Table I:** Demographics and fertility status and history of the sample participants (N = 49).

Demographic characteristics			
Biological sex, n (%) female		38	(77.6)
Age, mean (standard deviation), in years		39.16	(6.37)
Having a partner, n (%), yes		49	(100)
Age of the partner, mean (standard deviation), in years		40.51	(5.84)
Place of residency, n (%), city		47	(95.9)
Religious, n (%), yes		49	(100)
Fertility status/history			
Ever been pregnant from current partner, n (%), no ^1^		31	(63.3)
Having living children with current partner, n (%), yes ^2^		6	(33.3)
How long trying to conceive at this moment, mean (standard deviation), in years		7.61	(4.70)
Reason of current fertility problems, n (%)			
	Don’t know	18	(36.7)
	Male fertility problems	6	(12.2)
	Female fertility problems	17	(34.7)
	Male and female fertility problems	8	(16.3)

^1^ Men were asked if their current partner had been pregnant

^2^ Based on those who have been experiencing a pregnancy with their current partner

### Recruitment and procedure

Participants were recruited with the help of the Association of Childless Couples of Ghana (ACCOG), and a private hospital. Both were located in Accra (the capital of Ghana). People who were visiting the ACCOG or had a doctor’s appointment in the hospital for their fertility problems were informed about the study, and were asked to participate in it by handing over an information letter, in which the aims and method of the study was described. In the information letter we emphasised that participation was voluntary and the decision to participate or not would not have any consequences on their relationship with ACCOG nor on their access to infertility treatments. It was also stated on the letter that participants may skip any questions that raised discomforts, or could stop filling out the questionnaire at any time. It was also mentioned that if a participant objected to any question, that she or he had the opportunity to contact the investigators about this. Contact information of a counsellor was also included in the information letter (and also at the end of the questionnaire).

After signing the informed consent letter, participants were asked to fill out the questionnaire on the spot. They could also make an appointment with one of the research assistants to fill out the questionnaire at another time and/or at place. When filling out the questionnaire. research assistants were present in case participants had questions. The research protocol was approved by the Institutional Review Boards of the Noguchi Memorial Institute for Medical Research, University of Ghana and of the Faculty of Social and Behavioral Sciences, University of Amsterdam.

### Instruments

#### 

Data was collected with a paper-pencil questionnaire and included information on demographic characteristics and fertility status and history, experiences with stigmatisation of having fertility problems, coping with fertility problems, and quality of life in men and women experiencing fertility problems (henceforth called fertility quality of life). The scales that were used were all standardised instruments and were selected based on the criteria that they:(a) showed good psychometric properties in previous studies, (b) were available in English and (c) have been used in multiple countries, including non-Western parts of the world.

#### Demographic characteristics and fertility status and history.

The demographic characteristics measured included questions regarding biological sex, age, relationship status and age of partner, place of residency, and religion. Regarding the fertility status and history, information was obtained on whether the participant had ever been pregnant, whether the participant had ever conceived a child from their current partner, how long she or he had been trying to conceive, and what the reasons were for their current fertility problems.

#### Stigmatisation of having fertility problems.

Participants’ perceived experiences of stigmatisation as a result of having problems with conceiving or being childless was measured with a scale that has previously been developed and used by Donkor and Sandall (2007). Donkor and Sandall investigated the extent to which women in Southern Ghana, who were seeking infertility treatment, perceived themselves as being stigmatised because of having no children or having fertility problems. The scale included 3 items. One of the items, for example, was: “Because of my childlessness or fertility problems people treat me as inferior or look down upon me”. Participants were asked for each item whether or not this applied to them (0 = no, 1 = yes). A participant’s score on the stigmatisation scale was the sum of the responses of the 3 items. The Cronbach alpha obtained for this study was .90.

#### Coping with fertility problems.

In this current study a coping questionnaire was used that has previously been developed by Schmidt et al. (2005a, 2005b). This questionnaire specifically aimed to measure coping styles in relation to the specific stressor of having fertility problems, and included 4 subscales: (1) active-avoidance (4 items, e.g. “I avoid being with pregnant women or children”, Cronbach alpha = .58), (2) active-confronting (7 items, e.g. “I am showing my feelings, asking others for advice”, Cronbach alpha = .84), (3) passive-avoidance (2 items, e.g. “I feel that the only thing I can do is to wait”, Cronbach alpha = .67) , and (4) meaning- based (5 items, e.g. “I think about the fertility problem in a positive light/way”, Cronbach alpha = .85). Participants were asked to rate whether they had used these strategies on a 4-point Likert scale from 1 “not used” to 4 “used a great deal”. For each subscale the scores of the items were tabulated. The scores can be between 4 and 16 (active-avoidance), 7 and 28 (active-confronting), 2 and 8 (passive- avoidance), and 5 and 20 (meaning-based). A high score on these subscales reflects that a participant is using the coping strategy more often than a low score on the variable.

#### Fertility quality of life.

The total score of the Core Fertility Quality of Life (FertiQoL; Boivin et al., 2011) questionnaire was used to assess the impact of fertility problems on several domains (emotional, mind/body, relational and social) in one’s life. Participants were asked to answer on a 5-point Likert scale how much the items corresponded with their current thoughts and feelings (0 = “not at all”, 4 = “completely”). Items were reversed in such a way that a high score on a subscale means a high score on quality of life. The Core FertiQoL consists of 24 items divided in four subscales reflecting the separate domains. Examples of the items are: “Do you experience grief and/or feelings of loss about not being able to have a child” (emotional), “Is your attention/concentration impaired by thought of infertility” (mind/body), “Are you and your partner affectionate with each other even though you have fertility problems” (relational), and “Are you satisfied with the support you receive from friends with regard to your fertility problems” (social). Preliminary analyses showed moderate to high inter- correlations between the four subscales and therefore the total score of the Core FertiQol was used for the present study. In line with the instructions of the Core FertiQoL (available at http://sites.cardiff.ac.uk/fertiqol/scoring) participants’ raw scores were multiplied by 25/k, where k is the number of items (24). The score for the total scale can be between 0 and 100 (with a high score meaning a high level of quality of life).

### Statistical analyses

All statistical analyses were performed using IBM SPPS version 24. Descriptive analyses such as mean scores and standard deviations were conducted on the studied variables (experiences with stigmatisation of having fertility problems, coping with fertility problems, and fertility quality of life). Partial Pearson r correlations (controlled for biological sex) were calculated to investigate the associations between experiences with stigmatisation of having fertility problems, the four coping strategies, and fertility quality of life. To examine the mediation effect of the four coping strategies on the association between stigmatisation and quality of life, a bootstrapped mediation through the PROCESS macro (model 4) as developed by Hayes (2013) was used. This bootstrap method is recommended because it is suggested that this method is more appropriate than traditional approaches to mediation in models with more than one possible mediator (Hayes, 2009; Preacher and Hayes, 2008; Shrout and Bolger, 2002). Bootstrapping involves repeatedly (in this case, 10,000 times because of the limited sample size) randomly sampling cases based on the original data. We used a 95% confidence interval (CI) in these analyses. An assumed mediator variable is significant when the obtained CI does not contain the value 0 (Hayes, 2013). Biological sex was included in the analysis as a controlling variable. In this study the inclusion criterion to conduct the bootstrapped mediation analysis was that the (possible) mediator should be bivariate, significantly associated with the independent (experiences with stigmatisation of having fertility problems) and dependent variable (fertility quality of life), which was analysed with the partial Pearson r correlations.

## Results

### Descriptive analyses

Table II shows the means, standard deviations and (partial) correlations between the studied variables. Stigmatisation was negatively correlated with fertility quality of life, and positively with active-avoidance coping. Active-avoidance and active-confronting coping were positively related to each other. Active-confronting coping was also positively related to passive-avoidance and meaning-based coping. There was also a significant (positive) association between passive-avoidance and meaning-based coping. Fertility quality of life was significantly associated with only one of the four studied coping strategies, namely active- avoidance coping.

**Table II:** Mean (standard deviation), observed minimal and maximal scores, and (partial) correlations between studied variables.

		Observed min and max scores						
	M (SD)	Min	Max	1.	2.	3.	4.	5.
1. Stigmatisation	1.41 ( 1.37)	0.00	3.00	-				
2. Active-avoidance	7.96 (3.19)	3.00	15.00	.39 **	-			
3. Active-confronting	16.16 (5.94)	7.00	27.00	.29	.38 **	-		
4. Passive-avoidance	5.46 (2.22)	1.00	8.00	.06 ***	.14	.32 *	-	
5. Meaning-based	14.44 (04.37)	5.00	20.00	.09 ***	.28	.49 ***	.58 ***	
6. Quality of life	60.46 (20.14)	21.88	100.00	-.58 ***	-.43 **	-.24	-.14	-.15

Note: Absolute scores on these scales: Stigmatisation = 0-3, Active-avoidance: 3-15, Active confronting: 7-27, Passive-avoidance: 1-8, Meaning-based: 5-20, Quality of life: 21.00-100

*p < .05, **p < .01, ***p < .001

### Mediation of Coping Strategies on the Association between Stigmatisation for having Fertility Problems and Fertility Quality of Life

**Figure 1:**
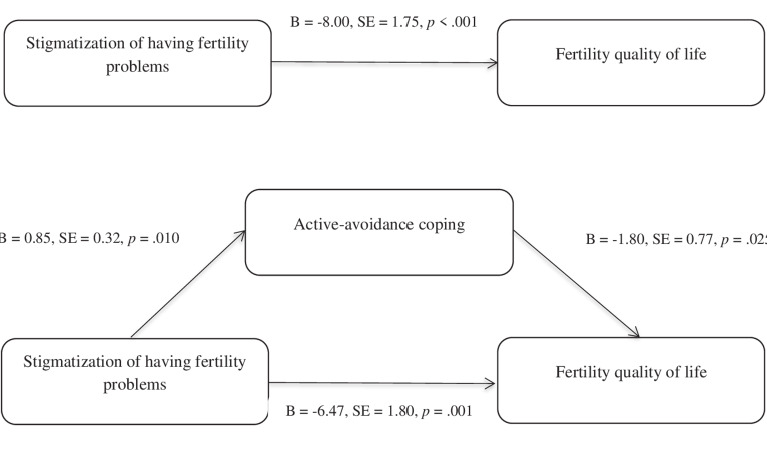
— Findings of the mediation analysis of active-avoidance coping as mediator of the association between stigmatization of ha ving fertility problems and fertility quality of life.

Active-avoidance coping was the only proposed mediator that met our inclusion criteria for the bootstrapping mediation analysis. Therefore, it was only assessed whether this coping style mediates the association between stigmatisation for having fertility problems and fertility quality of life, and evidence was found for this mediating effect of active-avoidance coping (B = -1.53, SE = .084, bootstrap 95%, CI = -3.41; -.19). Figure 1 displays the findings of this mediation and the unstandardized coefficients of each pathway (PROCESS Model 4). The overall model accounted for 35% of the variance in fertility quality of life, F (2,46) = 12.25, p < 0.001. Participants who showed higher levels of experiences of being stigmatized because of their fertility problems reported higher scores on active-avoidant coping (path a), and, in turn higher levels of this coping style predicted lower levels fertility quality of life (path b).As shown in Figure 1 (path c) the total direct effect of stigmatization for having fertility problems was a significant predictor of fertility quality of life, prior to entering the mediator variable, t = -4.56, p< .001, 95% CI = -11.53, - 4.47).The association between being stigmatised because of fertility problems and fertility quality of life remained significant after entering the mediator and the control variable (path c’), indicating that active-avoidance coping functions as a partial mediator.

## Discussion

In this study we looked at the possible relationship between experiences of stigmatisation and fertility quality of life in a cross section of Ghanaian women and men with fertility problems. Moreover, we looked at the possible mediating role of coping styles to better understand the underlying differences in how infertile Ghanaian women and men are affected in terms of their quality of life. The moderate average level of stigma and the wide diversity in stigma experiences confirms the findings of a larger sample of infertile women from the South of Ghana (Donkor and Sandall, 2007). Mean scores on fertility quality of life were sufficient, which appears to be quite similar to other countries where having children is highly valued (Maroufizadeh et al., 2016 (Iran); Priangga et al., 2016 (Indonesia); Sextyet al., 2016 (Jordan)). The respondents in our study used multiple coping strategies, of which passive avoidance and meaning based coping were used most often confirming previous findings (Donkor and Sandall, 2009).

Conforming to our hypothesis and confirming previous studies on the relationship between stigma and aspects of wellbeing (Alhassan et al., 2014; Donkor and Sandall, 2007; Naabet al., 2013), we found that participants with a higher score on stigmatisation showed lower scores on fertility quality of life (strong effect). Our hypothesis that this relationship would be indirect through the use of different coping strategies was only confirmed for the active-avoidance coping strategy, which partially mediated the association between stigma and quality of life: Experiencing stigma led to more active-avoidance coping strategies, which in turn led to a lower quality of life. No other indirect relationships were observed.

Some limitations need to be taken into account. First of all, we should be careful in drawing causal conclusions as we used a cross-sectional design in our study, whereas causality can be seen as a time-dependent concept. Although it is plausible, for example, that low levels of quality of life are a result of active-avoidance coping style, it might also be that problems related to quality of life might contribute to such a coping style. This can only be explored using a longitudinal study (Maxwell et al., 2011). Secondly, only a limited number of men were recruited, making it impossible to separately test the relationships between stigma and fertility quality of life and the mediating role of coping for men and women. Thirdly, all participants were recruited through a hospital and a patient support group, both located in an urban area. Although a large study in the south of Ghana found no significant difference between urban and non-urban settings regarding infertility related experienced stigma (Donkor and Sandall, 2007), the location might still have influenced possible relevant socio- demographic factors that influence infertility quality of life and coping, like being highly educated or having a professional career. These factors might also explain the diversity of stigma experiences and quality of life in our study. Furthermore, the variations might also be influenced by the rather diverse group of participants in our study in terms of age, length of infertility, mixture of primary and secondary infertility problems, and education (Alhassan et al., 2014).

The recruitment through a hospital and a patient support group might also have influenced coping and quality of life. All participants actively sought support (in terms of treatment, information, peer support or psychological counselling), which is a way of coping with infertility in itself. Additionally, seeking treatment might have given them a feeling of hope and agency, which may have had a positive influence on their quality of life (Naabet al., 2013). Based on studies of infertility support groups in the Western world (Stewart et al., 1992; Van Balen et al., 2001) and support groups for patients with other health issues, such as HIV/AIDS in the sub-Saharan African context (Gillett and Parr 2010; Moyer et al., 2013), a similar positive influence on quality of life might be expected in our study. Therefore, our findings might not be representative for the whole Ghanaian population with fertility problems.

Taking the aforementioned limitations into account, we would recommend further research to get a better picture of how the relationship between stigmatisation and fertility-related quality of life and the mediating role of coping strategies develop over time, using a longitudinal design, recruiting a bigger sample of Ghanaian men and women with fertility problems through multiple channels in multiple areas. A larger sample size might also enable us to distinguish possible differences in sex, education, work status, in the relationship between stigma and fertility-related quality of life and coping strategies, and the relationship between coping and quality of life. Insights into all these factors would give professionals working with people with fertility problems better guidelines to assist them.

In the meantime, our cross-sectional finding of the mediating role of the active-avoidance coping strategy in the relationship between experiencing stigma and fertility quality of life, suggests that it would be helpful if professionals would teach infertile men and women to use coping strategies other than the active-avoidance strategy in reaction to stigmatisation. Reducing the use of this strategy may increase quality of life. An even more powerful way to improve the fertility quality of life of Ghanaian men and women would be to reduce stigmatisation. Increasing access to proper information about fertility problems, its causes, and to fertility treatments may might not only lead to reduced infertility and increased timely responses, but also to less stigmatisation of fertility problems and childlessness (Gerrits et al., 2017; Inhorn and Patrizio, 2015; Ombelet, 2011).
